# Supplementary material for: Technological Improvements in the Genetic Diagnosis of Rett Syndrome Spectrum Disorders
Source: Int J Mol Sci. 2021 Sep 26;22(19):10375. doi: 10.3390/ijms221910375 (PMC8508637; doi:10.3390/ijms221910375)
Supplement: Supplementary file 1 [file ijms-22-10375-s001.zip › ijms-1341867-supplementary.pdf]

Table S1. Genes associated to RTT spectrum disorders in case reports from the HGMD-Professional database (June 2021) compared with OMIM phenotypes. The table shows the Gene Symbol, the general inheritance pattern (Inh) associated to each gene (AD = Autosomal Dominant, AR = Autosomal Recessive, XLD = X-Linked Dominant; XLR = X-Linked Recessive, NA = Not Available), the RTT spectrum cases reported in HGMD professional and their phenotypic description, as well as the phenotypes described in OMIM associated to each gene (NA = No phenotypes described in OMIM).

Gene Symbol	Inh	HGMD		OMIM		
		RTT spectrum phenotypes	N	Phenotypes	Inh	MIM#
ACTL6B	AD/AR	Rett syndrome, atypical	1	Developmental and epileptic encephalopathy 76	AR	618468
		Intellectual disability, ambulation deficits, language impairment, hypotonia, Rett-like stereotypies and facial dysmorphism	2	Intellectual developmental disorder with severe speech and ambulation defects	AD	618470
AGAP6	NA	Rett-like syndrome	1	NA		
ANKRD31	NA	Rett-like syndrome	1	NA		
ANXA11	AD	Rett-like syndrome	1	Amyotrophic lateral sclerosis 23	AD	617839
ATP6V0A1	NA	Rett syndrome, atypical	1	NA		
AUTS2	AD	Rett syndrome, atypical	1	Mental retardation, autosomal dominant 26	AD	615834
BRAF	AD	Rett syndrome, atypical	1	Cardiofaciocutaneous syndrome	AD	115150
				LEOPARD syndrome 3	AD	613707
				Noonan syndrome 7	AD	613706
BTBD9	NA	Rett-like syndrome	1	NA		

CACNA1A	AD	Rett syndrome, atypical	1	Developmental and epileptic encephalopathy 42	AD	617106
				Episodic ataxia, type 2	AD	108500
				Migraine, familial hemiplegic, 1	AD	141500
				Migraine, familial hemiplegic, 1, with progressive cerebellar ataxia	AD	141500
				Spinocerebellar ataxia 6	AD	183086
CACNA1D	NA	Rett-like syndrome	1	Primary aldosteronism, seizures, and neurologic abnormalities	AD	615474
				Sinoatrial node dysfunction and deafness	AR	614896
CACNA1G	AD	Rett-like syndrome	1	Spinocerebellar ataxia 42	AD	616795
				Spinocerebellar ataxia 42, early-onset, severe, with neurodevelopmental deficits	AD	618087
CACNA1I	NA	Rett-like syndrome	1	NA		
CAMK2B	NA	Rett-like syndrome	1	Mental retardation, autosomal dominant 54	AD	617799
CDKL5	XLD	*CDKL5* deficiency disorder	102	Developmental and epileptic encephalopathy 2	XLD	300672
		Rett syndrome	10			
		Rett syndrome, atypical	23			
		Rett syndrome, Hanefeld variant	4			
		Rett syndrome, variant, with infantile spasms	18			
		Encephalopathy, early-onset, and atypical Rett syndrome	3			
		Rett-like syndrome	1			
CHD4	NA	Rett syndrome	1	Sifrim-Hitz-Weiss syndrome	AD	617159

CHRNA5	NA	Rett-like syndrome	1	NA		
CLTC	AD	Rett-like syndrome	1	Mental retardation, autosomal dominant 56	AD	617854
COL4A1	AD	Rett-like syndrome	1	Angiopathy, hereditary, with nephripathy, aneurysms, and muscle cramps	AD	611773
				Brain small vessel disease with or without ocular anomalies	AD	175780
				Microangiopathy and leukoencephalopathy, pontine, autosomal dominant	AD	618564
CTNNB1	AD	Rett syndrome	1	Exudative vitreoretinopathy 7	AD	617572
		Rett-like syndrome	2	Neurodevelopmental disorder with spastic diplegia and visual defects	AD	615075
CUX2	NA	Rett-like syndrome	1	Developmental and epileptic encephalopathy 67	AD	618141
DMXL2	NA	Rett-like syndrome	1	?Deafnes, autosomal dominant 71	AD	617605
				?Polyendocrine-polyneuropathy syndrome	AR	616113
				Developmental and epileptic encephalopathy 81	AR	618663
DNMT3A	AD	Rett-like syndrome	1	Acute myeloid leukemia, somatic		601626
				Heyn-Sproul-Jackson syndrome	AD	618724
				Tatton-Brown-Rahman syndrome	AD	615879
EEF1A2	NA	Rett-like syndrome	1	Developmental and epileptic encephalopathy 33	AD	616409
				Mental retardation, autosomal dominant 38	AD	616393
EIF2B2	AR	Rett-like syndrome	1	Leukoencephalopathy with vanishing white matter	AR	603896
				Ovarioleukodystrophy	AR	603896

EIF4G1	AD	Rett-like syndrome	1	{Parkinson disease 18}	AD	614251
FAM151A	NA	Rett syndrome	1	NA		
FAT3	NA	Rett syndrome, atypical	1	NA		
FOXG1	AD	*FOXG1* syndrome	25	Rett syndrome, congenital variant	AD	613454
		Rett syndrome	36			
		Rett-like syndrome	10			
		Rett syndrome, atypical	6			
		Mental retardation, Rett-like	1			
		Rett syndrome features	1			
		Rett syndrome with hypoplastic hippocampus	1			
		Rett syndrome, congenital variant	1			
GABBR2	NA	Rett-like syndrome	1	Developmental and epileptic encephalopathy 59	AD	617904
				Neurodevelopmental disorder with poor language and loss of hand skills	AD	617903
GABRA1	AD	Rett-like syndrome	1	Developmental and epileptic encephalopathy 19	AD	615744
GABRB2	AD	Rett syndrome	1	Developmental and epileptic encephalopathy 92	AD	617829
		Rett syndrome, atypical	1			
GABRD	NA	Rett syndrome	1	{Epilepsy, generalized, with febrile seizures plus, type 5, susceptibility to}	AD	613060
				{Epilepsy, idiopathic generalized, 10}	AD	613060
				{Epilepsy, juvenile myoclonic, susceptibility to}	AD	613060

GABRG2	AD	Rett syndrome, atypical	1	Developmental and epileptic encephalopathy 74	AD	618396
				Epilepsy, generalized, with febrile seizures plus, type 3	AD	607681
				Febrile seizures, familial, 8	AD	607681
GRIA3	XLR	Rett-like syndrome	1	Intellectual developmental disorder, X-linked, syndromic, Wu type	XLR	300699
GRIN1	AD	Rett syndrome, atypical	1	Neurodevelopmental disorder with or without hyperkinetic movements and seizures, autosomal dominant	AD	614254
				Neurodevelopmental disorder with or without hyperkinetic movements and seizures, autosomal recessive	AR	617820
GRIN2A	AD	Rett syndrome, atypical	1	Epilepsy, focal, with speech disorder and with or without mental retardation	AD	245570
		Rett-like syndrome	1			
GRIN2B	AD	Rett syndrome, atypical	1	Developmental and epileptic encephalopathy 27	AD	616139
		Rett-like syndrome	1			
		Rett-like syndrome with encephalopathy	1	Intellectual developmental disorder, autosomal dominant 6, with or without seizures	AD	613970
		Atypical Rett syndrome & intractable epilepsy	1			
HCN1	NA	Rett-like syndrome	1	Developmental and epileptic encephalopathy 24	AD	615871
				Generalized epilepsy with febrile seizures plus, type 10	AD	618482
HDAC1	NA	Rett syndrome	1	NA		
HDAC8	XLD	Rett syndrome, atypical	1	Cornelia de Lange syndrome 5	XLD	300882
HECW2	NA	Rett-like syndrome	1	Neurodevelopmental disorder with hypotonia, seizures, and absent language	AD	617268

HNRNPU	AD	Developmental delay and Rett-like syndrome with acute encephalopathy and intractable epilepsy	1	Developmental and epileptic encephalopathy 54	AD	617391
HTT	AD	Rett-like syndrome	3	Huntington disease	AD	143100
				Lopes-Maciel-Rodan syndrome	AR	617435
IMPDH2	NA	Rett syndrome, atypical	1	[IMPDH2 enzyme activity, variation in]	NA	617995
IQGAP3	NA	Rett syndrome, atypical	1	NA		
IQSEC2	XLD	Rett syndrome	2	Mental retardation, X-linked 1/78	XLD	309530
		Rett syndrome, atypical	1			
		Rett syndrome features	1			
		Rett-like syndrome	1			
IRF2BPL	NA	Rett-like syndrome	1	Neurodevelopmental disorder with regression, abnormal movements, loss of speech, and seizures	AD	618088
ITPR1	AD/AR	Rett-like syndrome	1	Gillespie syndrome	AD/AR	206700
				Spinocerebellar ataxia 15	AD	606658
				Spinocerebellar ataxia 29, congenital nonprogressive	AD	117360
JMJD1C	AD	Rett syndrome	1	NA		
KCNA2	AR	Rett-like syndrome with infantile onset seizures	1	Developmental and epileptic encephalopathy 32	AD	616366
KCNB1	AD	Rett syndrome, atypical	1	Developmental and epileptic encephalopathy 26	AD	616056
KCNJ10	AR	Rett syndrome	1	Enlarged vestibular aqueduct, digenic	AR	600791
				SESAME syndrome	AR	612780

KCNQ2	AD	Rett syndrome	1	Developmental and epileptic encephalopathy 7	AD	613720
KCNQ2	AD	Rett syndrome	1	Myokymia	AD	121200
KCNQ2	AD	Rett syndrome	1	Seizures, benign neonatal, 1	AD	121200
KIF1A	AR	Rett syndrome	2	NESCAV syndrome	AD	614255
KIF1A	AR	Rett syndrome	2	Neuropathy, hereditary sensory, type IIC	AR	614213
KIF1A	AR	Rett-like syndrome	1	Spastic paraplegia 30, autosomal dominant	AD/AR	610357
KIF1A	AR	Rett-like syndrome	1	Spastic paraplegia 30, autosomal recessive	AD/AR	610357
KIF4B	NA	Rett-like syndrome	1	NA		
LAMB2	AR	Rett syndrome	1	Nephrotic syndrome, type 5, with or without ocular abnormalities		614199
LAMB2	AR	Rett syndrome	1	Pierson syndrome	AR	609049
LRRC40	NA	Rett syndrome	1	NA		
MAST1	NA	Rett syndrome, atypical	1	Mega-corpus-callosum syndrome with cerebellar hypoplasia and cortical malformations	AD	618273
MAST3	NA	Rett-like syndrome	1	NA		

MECP2	XLD	Rett syndrome	719	{Autism susceptibility, X-liked 3}	XL	300496
				Encephalopathy, neonatal severe	XLR	300673
		Rett syndrome, atypical	22	Mental retardation, X-linked syndromic, Lubs type	XLR	300260
		Rett syndrome, preserved speech variant	5	Mental retardation, X-linked, syndromic 13	XLR	300055
		Rett-like syndrome	3	Rett syndrome	XLD	312750
		Rett syndrome, variant	1	Rett syndrome, atypical	XLD	312750
		Rett syndrome, variant, with autism	1	Rett syndrome, preserved speech variant	XLD	312750
MEF2C	AD	Rett syndrome	4	Chromosome 5q14.3 deletion syndrome	AD	613443
		Rett like features	1			
		Rett like intellectual disability	1	Mental retardation, stereotypic movements, epilepsy, and/or cerebral malformations	AD	613443
		Rett/rett-like syndrome and/or non-syndromic intellectual disability	1			
MEIS2	NA	Rett syndrome, atypical	1	Cleft palate, cardiac defects, and mental retardation	AD	600987
MGRN1	NA	Rett-like syndrome	1	NA		
NALCN	AD/AR	Rett-like syndrome	1	Congenital contractures of the limbs and face, hypotonia, and developmental delay	AD	616266
				Hypotonia, infantile, with psychomotor retardation and characteristic facies 1	AR	615419
NCOR2	NA	Rett syndrome, atypical	1	NA		
		Rett-like syndrome	2			
NR2F1	AD	Rett-like syndrome	1	Bosch-Boonstra-Schaaf optic atrophy syndrome	AD	615722
NTNG1	NA	Rett syndrome	1	NA		

NTNG2	NA	Rett-like syndrome with areflexia	1	Neurodevelopmental disorder with behavioral abnormalities, absent speech, and hypotonia	AR	618718
OSBP	NA	Rett-like syndrome	1	NA		
PDE2A	NA	Rett syndrome, atypical	1	Intellectual developmental disorder with paroxysmal dyskinesia or seizures	AR	619150
PDHA1	XLD	Rett syndrome, atypical	1	Pyruvate dehydrogenase E1-alpha deficiency	XLD	312170
PDLIM7	NA	Rett-like syndrome	1	NA		
PPT1	AR	Rett syndrome, atypical	1	Ceroid lipofuscinosis, neuronal, 1	AR	256730
PTPN4	NA	Rett-like syndrome	1	NA		
RHOBTB2	NA	Rett-like syndrome	5	Developmental and epileptic encephalopathy 64	AD	618004
RRN3	NA	Rett-like syndrome	1	NA		
SATB2	AD	Rett-like syndrome	2	Glass syndrome	AD	612313
SCN1A	AD	Rett syndrome	2	Developmental and epileptic encephalopathy 6B, non-Dravet	AD	619317
SCN1A	AD	Rett syndrome	2	Dravet syndrome	AD	607208
SCN1A	AD	Rett syndrome	2	Epilepsy, generalized, with febrile seizures plus, type 2	AD	604403
SCN1A	AD	Rett-like syndrome	1	Febrile seizures, familial, 3A	AD	604403
SCN1A	AD	Rett-like syndrome	1	Migraine, familial hemiplegic, 3	AD	609634
SCN2A	AD	Rett syndrome	1	Developmental and epileptic encephalopathy 11	AD	613721
SCN2A	AD	Rett syndrome	1	Episodic ataxia, type 9	AD	618924
SCN2A	AD	Rett syndrome	1	Seizures, benign familial infantile, 3	AD	607745

SCN8A	AD	Rett syndrome, atypical	1	?Myoclonus, familial, 2	AD	618364
				Cognitive impairment with or without cerebellar ataxia	AD	614306
		Rett syndrome features	1	Developmental and epileptic encephalopathy 13	AD	614558
				Seizures, benign familial infantile, 5	AD	617080
SDHA	AD	Rett syndrome	1	Cardiomyopathy, dilated, 1GG	AR	613642
				Mitochondrial complex II deficiency, nuclear type 1	AR	252011
				Neurodegeneration with ataxia and late-onset optic atrophy	AD	619259
				Paragangliomas 5	AD	614165
SEMA6B	NA	Rett-like syndrome	1	Epilepsy, progressive myoclonic, 11	AD	618876
SHANK3	AD	Rett syndrome	2	{Schizophrenia 15}	AD	613950
		Rett syndrome, atypical	1	Phelan-McDermid syndrome	AD	606232
		Rett-like syndrome	2			
SLC35A2	XLD	Rett-like syndrome	1	Congenital disorder of glycosylation, type IIm	SMo, XLD	300896
SLC39A13	AR	Rett syndrome, atypical	1	Ehlers-Danlos syndrome, spondylodysplastic type, 3	AR	612350
SLC6A1	AD	Rett syndrome	1	Myoclonic-atonic epilepsy	AD	616421
		Rett-like syndrome	2			
SMARCA1	NA	Rett syndrome	1	NA		

SMC1A	XLD	Rett syndrome	1	Cornelia de Lange syndrome 2	XLD	300590
		Rett-like syndrome	2	Developmental and epileptic encephalopathy 85, with or without midline brain defects	XLD	301044
STXBP1	AD	Rett syndrome	1	Developmental and epileptic encephalopathy 4	AD	612164
		Rett syndrome, atypical	1			
		Rett-like syndrome	2			
		Rett syndrome features	1			
SYNGAP1	AD	Rett-like syndrome	1	Mental retardation, autosomal dominant 5	AD	612621
TBL1XR1	AD	Rett-like syndrome	1	Mental retardation, autosomal dominant 41	AD	616944
				Pierpont syndrome	AD	602342
TCF4	AD	Rett syndrome, atypical	2	Corneal dystrophy, Fuchs endothelial, 3	AD	613267
		Rett syndrome, variant / Pitt-Hopkins syndrome	1			
		Rett-like syndrome	1	Pitt-Hopkins syndrome	AD	610954
TRRAP	AD	Rett syndrome, atypical	1	?Deafness, autosomal dominant 75	AD	618778
				Developmental delay with or without dysmorphic facies and autism	AD	618454
UBE3A	AD	Rett-like syndrome	1	Angelman syndrome	AD	105830
USP8	NA	Rett syndrome, atypical	1	Pituitary adenoma 4, ACTH-secreting, somatic	NA	219090
WDR45	XLD	Rett syndrome	2	Neurodegeneration with brain iron accumulation 5	XLD	300894
		Rett syndrome, atypical	2			
		Rett-like syndrome	2			
ZNF620	NA	Rett-like syndrome	1	NA		
